# Complications of Ultrasound-guided Liver Biopsy at a Tertiary Care Hospital in Pakistan: An Audit

**DOI:** 10.7759/cureus.5811

**Published:** 2019-10-01

**Authors:** Basit Salam, Fatima Mubarak, Yusra Waheed, Noman Khan

**Affiliations:** 1 Radiology, Aga Khan University Hospital, Karachi, PAK; 2 Diagnostic Radiology, Aga Khan University Hospital, Karachi , PAK

**Keywords:** liver biopsy, complications of liver biopsy, aga khan university hospital, liver biopsy in pakistan, ultrasound-guided liver biopsy, image-guided liver biopsy, us-guided liver biopsy

## Abstract

Introduction

Liver diseases account for two million deaths per year worldwide, half of which are attributed to complications of cirrhosis. Liver conditions have wide-ranging serological findings and imaging appearances and may require biopsy for a definitive diagnosis. Despite ultrasound (US) guidance, liver biopsy is an invasive procedure and the expected benefit must outweigh risks involved.

Objective

The purpose of the audit was to calculate complication rates of US-guided liver biopsy and summarize institutional data pertaining to the procedure.

Materials and Methods

The audit was performed at Aga Khan University Hospital, Karachi, Pakistan. All consecutive patients undergoing liver biopsy from February 2017 - February 2018 were included. Medical records of patients were reviewed for complications of liver biopsy.

Results

The study population consisted of 157 adult and 21 pediatric patients. Complications were encountered in nine patients (5%), minor complications in seven (4%) and major complications in two (1.1%). Among the minor complications, haematoma formation was noted in four patients (2.2%), minor abdominal pain in two patients (1.1%), and minor hemorrhage during the procedure in one patient (0.5%). Minor complications were seen more frequently in pediatric (14%) patients as compared to adults (3.8%). One patient developed a major hemorrhage (> 2 g/dl drop in hemoglobin (Hb)), and another patient developed severe vasovagal hypotension. There was no mortality in the study population resulting from complications of the liver biopsy. The audit standards set were met for all parameters, except major hemorrhage (< 0.5%) which was narrowly missed (0.56%).

Conclusion

US-guided liver biopsy at our institution has a good safety profile with complication rates within the expected range. Departmental practices are compliant with established practices and guidelines.

## Introduction

Liver diseases account for approximately two million deaths per year worldwide, half of which are attributed to complications of cirrhosis. Cirrhotic liver disease is 11th and liver cancer is 16th most common cause of death globally [1-2]. Liver diseases are wide-ranging with varying clinical, serological, and imaging findings. Although serological markers and imaging are routinely ordered in the evaluation of liver diseases, these are no substitute for a liver biopsy which provides a definitive diagnosis [3].

A liver biopsy procedure is preferably performed with ultrasound (US) guidance in which multiple passes may be acquired to obtain a diagnostic sample. US-guided liver biopsy is a cost-effective procedure owing to its high specimen adequacy rate and low complication rate [4]. Despite real-time imaging guidance with US, a liver biopsy is an invasive, non-vascular interventional procedure, and therefore, the benefits of biopsy must outweigh the risks of complications [5].

Data regarding the safety profile of liver biopsy in local healthcare institutions is lacking. The objective of our study was to determine the safety profile of liver biopsy procedures at our institution. 

## Materials and methods

The audit was performed in the Department of Radiology at the Aga Khan University Hospital, Pakistan. The nature of the study was retrospective and was approved by the institutional ethics review committee with a waiver of consent. All consecutive patients undergoing US-guided liver biopsy during the study period of February 2017 - February 2018 were included. Data were retrieved using the radiology information system (RIS), hospital information system (HIS) and picture archiving and communications system (PACS). Audit standards were set according to the findings of the United Kingdom (UK) National Audit Evaluating Image-guided or Image-assisted Liver Biopsy, Parts I and II, as listed in Table 1 [6-7].

**Table 1:** Audit Standards

Audit Standards	
Completed consent form	100%
Documented post-procedural instructions	100%
Specimen adequacy	> 98%
Normal baseline coagulation	100%
Minor pain	< 30%
Significant hemorrhage	< 0.5%
Vasovagal hypotension	< 3%
Severe pain	< 3%
Puncture of another organ	< 0.1%
Death	< 0.1%
Hemobilia	< 0.1%

Data items that were collected are listed in Table 2. Complications related to liver biopsy were broadly classified into major and minor complications. Major complications included significant hemorrhage (> 2 g/dl), severe hypotension, organ perforation, hemobilia, and death. The minor complications included minor pain, hypotension, and minor hemorrhage [7]. 

**Table 2:** Data Items Collected FAST: focused assessment with sonography for trauma

Data items collected
Patient demographics
Referrer
Clinical information
Coagulation profile
Location of biopsy
Needle size
History of repeat biopsy
Complication(s) documented
Postprocedure FAST
Histopathology report
Documented post-procedural instructions

A summary of departmental practices is listed in Table 3. 

**Table 3:** Summary of Departmental Practices Tru-Cut® biopsy needle (Merit Medical Systems, Jordan, UT) FAST: focused assessment with sonography for trauma; INR: international normalized ratio

Summary of departmental practices
Prior imaging is reviewed by the radiologist at the time of appointment and prior to biopsy
Coagulation profile is obtained as per departmental policy for invasive procedures (INR ≤ 1.5 and platelet count ≥ 75 × 109/ L, within the same week)
Informed consent obtained as per hospital policy
Sedation required in all pediatric patients
Pre-procedure sonographic evaluation for the presence of ascites, approach determination, and pre-marking of the biopsy site. Procedure postponed if ascites is present
Determination of the size of Tru-Cut® biopsy needle is made by the performing radiologist
Biopsy is performed using a coaxial technique or non-coaxial technique and multiple passes may be made
Post-procedure, patients are observed for 4 hours with regular vitals monitoring. Appropriate analgesia is given for pain
Post-procedure FAST is performed within 2 hours to assess for hemoperitoneum
Stable patients with no major pain and without hemoperitoneum are discharged with instructions

Data were entered into a worksheet (Microsoft® Office Excel 2013; Microsoft® Corp., Redmond, WA) and percentage frequency of major and minor complications were calculated.

## Results

The final study population consisted of 178 patients. An even gender distribution was noted, with 48.9% male and 51% female patients. The mean age of the study population was 47.63 years (range: 1 - 82 years). 

Complications were encountered in nine (5%) patients, minor in seven patients (4%) and major in two patients (1.1%). Of the minor complications, hematoma formation was noted in four patients (2.2%), minor abdominal pain in two patients (1.1%), and minor haemorrhage during the procedure in one patient (0.5%). In patients who had major complications, one patient had a significant intraperitoneal hemorrhage (> 2 g/dl drop in hemoglobin) and one patient developed severe vasovagal hypotension. These are summarized in Table 4. 

**Table 4:** Post-biopsy Complications

Complication	Frequency (%)
Overall	5 (9/178)
Minor	3.9 (7/178)
Major	1.1 (2/178)

There was no mortality recorded in the study population resulting from complications of the biopsy procedure.

Compared to the adult population, the complication rate was slightly higher in pediatric patients (14%) as compared to adults (3.8%). These were, however, minor complications and no major complication was encountered in any pediatric patient. 

A targeted biopsy was performed in 164 patients (92%), with right lobe lesions biopsied in 157 patients and left lobe lesions in seven patients. A non-targeted biopsy was performed in 14 patients (7.8%), and a biopsy specimen was obtained from the right lobe in all those cases. 

The majority of patients were referred from service lines within the institution. Seventy-seven patients (43.2%) were referred from outpatient services and 52 (29.2%) from inpatient services. Forty-nine patients (27.5%) were referred from outside clinics. 

On radiology request forms for biopsy, adequate clinical information was provided in 177 (99%) patients. In one patient, the field for clinical information on the radiology request form was left blank.

Both 18 gauge (G) and 16 G needles were used for biopsy. An 18 G needle was used in 109 (61.2%) procedures and a 16 G in 69 (38.7%) procedures.

Based on the biopsy specimen, a final histopathological diagnosis was reached in 170 patients (95%). A sampling error was noted in one patient (0.56%), resulting in a repeat biopsy. Histopathological findings were inconclusive in one patient (0.56%). The histopathology records of four patients (2.2%) were not available. The biopsy specimens of two patients (1.1%) were sent to outside institutions and these records were not available. 

The audit standards set were met for all parameters, except for significant haemorrhage, in which the target was narrowly missed (Table 5).

**Table 5:** Compliance with Audit Standards

Compliance with Audit Standards			
Parameter	Target (%)	Rate achieved (%)*	Was target met?
Completed consent form	100	100 (178/178)	Yes
Post-procedural instructions	100	100 (178/178)	Yes
Specimen adequacy	> 98	99 (171/172)	Yes
Minor pain	< 30	1.1 (2/178)	Yes
Severe pain	< 3	0% (0/178)	Yes
Significant hemorrhage	< 0.5	0.56 (1/178)	No
Vasovagal hypotension	< 3	0.56 (1/178)	Yes
Puncture of another organ	< 0.1	0% (0/178)	Yes
Hemobilia	< 0.1	0% (0/178)	Yes
Death	< 0.1	0% (0/178)	Yes

## Discussion

In this study, records of 178 patients were retrospectively evaluated for complications of ultrasound-guided liver biopsy. Liver biopsy is an invasive procedure with associated risks that must be weighed against the benefit of obtaining histology. The rate of complications must be minimized to acceptable safety margins [5].

In our department, all liver biopsy procedures are performed with image guidance by experienced radiologists. This is safer than image-assisted liver biopsy in which the biopsy site is pre-marked without real-time needle visualization during the procedure. Audit standards set for procedural aspects, diagnostic adequacy, and minor complications were met. Audit standards were also met for major complications, except for significant hemorrhage in which it was narrowly missed. No death was reported after the liver biopsy in our study. 

Overall complications occurred in nine patients (5%); seven complications (3.9%) were minor and two complications (1.1%) were major. In patients who developed major complications, one patient had a significant hemorrhage (> 2g/dL drop in hemoglobin) and another developed severe hypotension.

The patient with major hemorrhage had been referred from an outside clinic. He had a diagnosis of adenocarcinoma of the lung with Stage IV disease. His pre-procedure platelet count was 34 × 109/ L, and he had received platelet transfusions prior to biopsy. A repeat platelet count after transfusion had not been obtained. The international normalized ratio (INR) checked more than one week before the biopsy was > 1.5, which had been corrected one day prior to the procedure. The underlying deranged coagulation profile may have contributed to the adverse outcome seen in this patient. The immediate post-biopsy focused assessment with sonography for trauma (FAST) examination was positive in this patient; however, the patient was discharged as free fluid was mild and the amount remained unchanged over serial FAST examinations. The patient presented in the emergency room (ER) two hours after discharge with severe hypotension and Type I respiratory failure. There was a drop in the hemoglobin from 12 mg/dL pre-procedure to 7.4 mg/dL post-procedure. A computed tomography (CT) scan revealed hemorrhagic intraperitoneal free fluid in the perihepatic and perisplenic regions. The patient was managed conservatively, received intravenous fluids in the ER, and was admitted. He was discharged after 12 days in stable condition. The patient presented in the ER again three months later with severe respiratory distress and expired due to a massive pulmonary embolism.

Another patient with a major complication was an inpatient who developed severe hypotension after the biopsy. The INR and platelet counts were within normal limits. A CT scan revealed a small perihepatic hematoma and mild ascites. This patient was also managed conservatively and was later discharged in stable condition. 

A baseline of INR ≤ 1.5 and platelet count ≥ 75 × 109/ L is required for all invasive procedures as per departmental protocol. The INR and platelet counts of all patients were checked within the same week.

The complication rate did not increase when the biopsy was performed with a larger needle size. When a 16 G needle was used, complications occurred in two patients (2.8%). In contrast, complications occurred in seven patients (6.4%) when an 18 G needle was used. This is interesting as a larger needle size is usually associated with a higher risk of bleeding [8]. The slightly higher complication rate for an 18 G needle may be explained by its relatively more frequent use.

## Conclusions

US-guided liver biopsy performed at our institution has a good safety profile with low complication rates. Departmental policies and practices are compliant with established guidelines.
